# The principle of metal-zeolite catalyzing alkane dehydrogenation

**DOI:** 10.1093/nsr/nwaf446

**Published:** 2025-10-21

**Authors:** Haibo Zhu, Xiaojun Bao

**Affiliations:** College of Chemical Engineering, Fuzhou University, China; Qingyuan Innovation Laboratory, China

Alkanes are highly stable molecules that are challenging to activate and convert due to their low reactivity [1]. Recently, metal-zeolites have emerged as efficient catalysts for alkane dehydrogenation, sparking widespread interest and research [2−6]. While current studies primarily focus on the development of high-performance catalysts, a fundamental understanding of how the metal center and zeolite microenvironment interact and evolve to enable efficient alkane dehydrogenation under operational conditions remains elusive. This gap arises from the inherent structural complexity and flexibility of metal-zeolites during catalysis, highlighting the need for a comprehensive approach that integrates materials science and catalytic science for developing novel alkane conversion catalysts.

**Figure 1.**
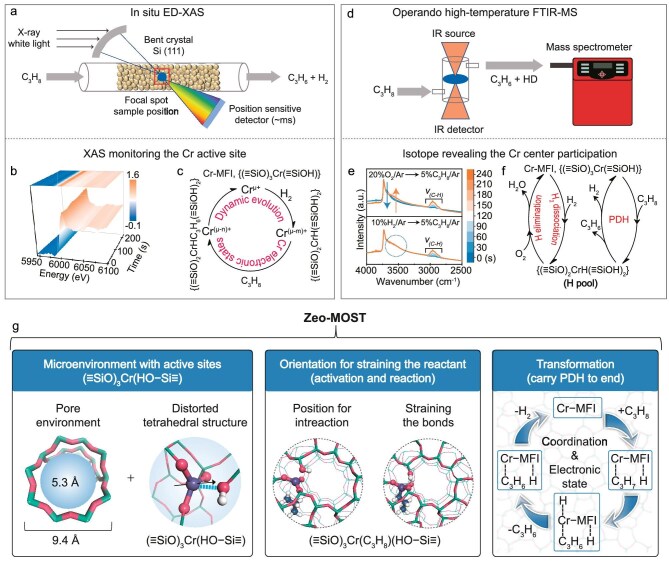
(a) Schematic diagram of the *in-situ* time-resolved energy-dispersive X-ray absorption spectroscopy (ED-XAS) devices. (b) *In situ* Cr K-edge XANES spectra of Cr-MFI during PDH. Reaction conditions: 580^o^C, 5%C_3_H_8_/Ar, 20 mL min^−1^. (c) Schematic illustration of the dynamic evolution of the Cr electronic states in H_2_ and C_3_H_8_ atmospheres. (d) Schematic diagram of the *in situ* high-temperature FTIR spectroscopy devices with on-line mass spectrometer. (e) *In situ* high-temperature FTIR spectra of Cr-MFI in 20%O_2_/Ar and switching transients to 5%C_3_H_8_/Ar (top), 10%H_2_/Ar and switching transients to 5%C_3_H_8_/Ar (down). (f) Schematic illustration of the evolution of hydrogen network in different atmospheres. (g) Zeo-MOST decipher metal-zeolite catalysis for alkane dehydrogenation. Microenvironment (M) with active site orientation (O) leading to C–H bond straining (S) and propane transformation (T). Adapted with permission from ref. [7].

In a recent study, Zhongmin Liu’s group at Dalian Institute of Chemical Physics, Chinese Academy of Sciences, reported a significant breakthrough in zeolite material science and catalysis, particularly concerning Lewis acid catalysis from isolated metallic centers within a zeolite microenvironment for alkane activation and dehydrogenation [7]. To achieve efficient alkane conversion and accurately and comprehensively elucidate the catalysis of metal-zeolites in alkane activation and conversion, they developed a Cr-MFI metal-zeolite model catalyst that demonstrates exceptional performance in propane dehydrogenation (PDH). Utilizing *in-situ* synchrotron radiation X-ray absorption spectroscopy, they achieved real-time observation of changes in the metal’s electronic state with millisecond resolution (Fig. 1a–c), confirming the electron transfer process. Additionally, they innovated high-temperature synchrotron radiation *in-situ* infrared technology to realize *in situ* observation of the transfer of hydrogen species to adjacent oxygen atoms during C–H bond dissociation at 580°C, successfully eliminating thermal signal interference (Fig. 1d–f).

By combining theoretical calculations and isotope labeling, this group fully elucidated the fundamental principles governing C–H bond dissociation and H–H bond formation in metal-zeolite catalysis. They found that the metal species incorporated into the zeolite framework form a unique twisted tetrahedral coordination structure. In this configuration, the zeolite microenvironment enhances the activity of the metal center for propane activation and enables the adjacent oxygen atoms to accept and transfer hydrogen. Building on these findings, they proposed the Zeo-MOST concept to elucidate the powerful and targeted catalytic performance of metal-zeolites in alkane dehydrogenation, particularly in the PDH reaction (Fig. 1g). The zeolite microenvironment (M) and the active metal site orientation (O) play a pivotal role in straining (S) the C–H bonds in propane, ultimately leading to the efficient transformation (T) of propane into the target product. The dynamic evolution of the Cr-center, in conjunction with the zeolite microenvironment, orchestrates and fine-tunes each step of the propane conversion process. The continuous operation of the Zeo-MOST mechanism ensures the highly efficient completion of the PDH reaction in the catalytic cycle. This concept not only provides an innovative and deep understanding of the core mechanisms of metal-zeolite catalysis but also offers valuable insights for the rational design and optimization of these catalysts for industrial applications, establishing a rigorous principle-based theoretical foundation to guide their development.
